# 4DMRI-based investigation on the interplay effect for pencil beam scanning proton therapy of pancreatic cancer patients

**DOI:** 10.1186/s13014-019-1231-2

**Published:** 2019-02-07

**Authors:** Kai Dolde, Ye Zhang, Naved Chaudhri, Christian Dávid, Marc Kachelrieß, Antony John Lomax, Patrick Naumann, Nami Saito, Damien Charles Weber, Asja Pfaffenberger

**Affiliations:** 1 Medical Physics in Radiation Oncology, German Cancer Research Center (DKFZ), Im Neuenheimer Feld 280, 69120 Heidelberg, Germany; 2 National Center for Radiation Research in Oncology (NCRO), Heidelberg Institute for Radiooncology (HIRO), Im Neuenheimer Feld 280, 69120 Heidelberg, Germany; 3 Department of Physics and Astronomy, Heidelberg University, Im Neuenheimer Feld 226, 69120 Heidelberg, Germany; 4 Center for Proton Therapy, Paul Scherrer Institute (PSI), 5232 Villigen-PSI, Switzerland; 5 Heidelberg Ion-Beam Therapy Center (HIT), Im Neuenheimer Feld 450, 69120 Heidelberg, Germany; 6 X-Ray Imaging and Computed Tomography, German Cancer Research Center (DKFZ), Heidelberg, Germany; 7 Department of Physics, ETH Zurich, 8092 Zurich, Switzerland; 8 Department of Radiation Oncology, University Clinic Heidelberg, Im Neuenheimer Feld 672, 69120 Heidelberg, Germany

**Keywords:** Proton therapy, Pencil beam scanning, Interplay effect, 4D-MRI, 4D dose calculations, Pancreatic cancer

## Abstract

**Background:**

Time-resolved volumetric magnetic resonance imaging (4DMRI) offers the potential to analyze 3D motion with high soft-tissue contrast without additional imaging dose. We use 4DMRI to investigate the interplay effect for pencil beam scanning (PBS) proton therapy of pancreatic cancer and to quantify the dependency of residual interplay effects on the number of treatment fractions.

**Methods:**

Based on repeated 4DMRI datasets for nine pancreatic cancer patients, synthetic 4DCTs were generated by warping static 3DCTs with 4DMRI deformation vector fields. 4D dose calculations for scanned proton therapy were performed to quantify the interplay effect by CTV coverage (v95) and dose homogeneity (d5/d95) for incrementally up to 28 fractions. The interplay effect was further correlated to CTV motion characteristics. For quality assurance, volume and mass conservation were evaluated by Jacobian determinants and volume-density comparisons.

**Results:**

For the underlying patient cohort with CTV motion amplitudes < 15 mm, we observed significant correlations between CTV motion amplitudes and both the length of breathing cycles and the interplay effect. For individual fractions, tumor underdosage down to v95 = 70% was observed with pronounced dose heterogeneity (d5/d95 = 1.3). For full × 28 fractionated treatments, we observed a mitigation of the interplay effect with increasing fraction numbers. On average, after seven fractions, a CTV coverage with 95–107% of the prescribed dose was reached with sufficient dose homogeneity. For organs at risk, no significant differences were found between the static and accumulated dose plans for 28 fractions.

**Conclusion:**

Intrafractional organ motion exhibits a large interplay effect for PBS proton therapy of pancreatic cancer. The interplay effect correlates with CTV motion, but can be mitigated efficiently by fractionation, mainly due to different breathing starting phases in fractionated treatments. For hypofractionated treatments, a further restriction of motion may be required. Repeated 4DMRI measurements are a viable tool for pre- and post-treatment evaluations of the interplay effect.

## Introduction

Pancreatic cancer is one of the leading causes of cancer deaths and shows a low 5-year survival rate of 5–20%, depending on stage at diagnosis [1, 2]. Currently, surgery remains the only potential possibility for curative treatment in localized disease, whereas radiotherapy (RT) and chemotherapy or combinations of both are used to improve patient survival in unresectable locally advanced stage.

Nevertheless, new RT techniques are considered more effective [3].

RT treatments of the pancreas are challenging due to the limited tolerance doses of adjacent organs at risk (OARs). While intensity-modulated radiation therapy (IMRT) is already able to decrease the doses to the GI-tract and the liver compared to 3D-conformal RT [4], particle therapy is a more promising modality to further reduce OAR toxicity by making use of the physical characteristics of the “Bragg-peak” [5]. Several encouraging preliminary results have been reported from clinical trials using carbon ions [6, 7] or proton radiotherapy with concomitant chemotherapy [8, 9].

However, the advantages of a high spatial accuracy in particle therapy are partly counteracted by various types of uncertainties, especially in the presence of intrafractional respiration-induced organ motion, which may lead to density changes in the proton beam paths. These changes are known to potentially cause heterogeneous dose distributions in the tumor and over- or undershooting of the target [10, 11].

For pencil beam scanning (PBS) particle therapy [12], the resulting interplay effects [13] between beam and tumor are considerable. Interplay effects have been investigated for different anatomical sites, such as lung [14] and liver tumors [15, 16], and various motion management techniques (e.g. rescanning, gating, breath-hold) have been proposed and analyzed [17–19] to mitigate the impact of motion. Nevertheless, compared to lung tumors whose motion can reach amplitudes of up to 53 mm [20], pancreatic motion is comparable to liver motion and therefore smaller due to the increased distance to the lung region [21]. However, since stomach and bowel filling influence the pancreas position in general, day-to-day pancreatic motion variations may occur.

Therefore, in this study it was the aim to evaluate the interplay effect using repeated time-resolved volumetric magnetic resonance imaging (4DMRI) for PBS proton therapy of pancreatic cancer. This approach allowed us to take into account both intra- and interfractional patient-specific organ motion and motion variations without exposing the patients to any additional imaging dose. We performed 4D dose calculations to quantify the interplay effect. The dosimetric analysis was based on synthetic 4DCTs, which were generated by warping static patient CTs by means of deformation vector fields, extracted from repeated 4DMRI data sets of patients.

Such an approach was initially developed for liver [22] and then utilized for one pancreas case in a proof-of-principle study [23]. In this previous methodical paper, we showed the feasibility of the synthetic 4DCT approach for pancreatic cancer, demonstrated the method by evaluating the statistical evolution of the interplay effect as a function of treatment fractionation and reported on the mitigation of the interplay effect by fractionation for one example pancreas case. However, due to the lack of statistics, it was not possible to quantitatively conclude on the dosimetric impact of pancreas motion, the number of required treatment fractions for an acceptable mitigation, the impact of the interplay effect on organs at risk and its correlations to tumor motion characteristics. In another recent study, which investigated the interplay effect for a cohort of 14 pancreatic cancer patients based on single 4DCTs for proton or carbon ion treatments [24], it was only possible to report the magnitude of interplay effect in a single fraction. Consequently, the efforts of the two last-mentioned studies were combined here, to evaluate 9 pancreatic cancer patients through the novel 4DMRI procedures with up to six repeated 4DMRI data sets per patient. Such repeated 4D data sets enabled a longitudinal analysis of the interplay effect as a function of the number of treatment fractions. In this study, we first aimed to quantify the patient-specificity of both day-to-day motion variations and the fractionation-induced interplay mitigation on the planned target dose and OARs. Second, we statistically determined the interplay impact on organs at risk and evaluated correlations and factors that influence the magnitude of the interplay effect. Finally, we investigated the clinical applicability of our approach with respect to a possible 4DMRI-based pre-treatment estimation of the evolution of the interplay effect along the treatment course.

## Material and methods

### Patient data

This study comprises a cohort of 9 pancreatic cancer patients, (6 females, 3 males) with a mean age of 65.9 years (50–82 years), who received either proton or carbon ion beam therapy at the Heidelberg Ion-Beam Therapy Center (HIT). Written informed consent for proton/carbon treatment and repeated MR imaging for positioning control as an individual treatment approach was obtained from all patients. For each patient, a 3D treatment planning CT, acquired under free breathing, was available on which the gross tumor volume (GTV), the clinical target volume (CTV), the internal target volume (ITV), the planning target volume (PTV) and the OARs were defined [25]. The GTV and CTV were delineated by a radiation oncologist, whereas the ITV was generated by means of the union of all CTVs within all breathing phases of an acquired 4DCT measurement. The PTV was defined by adding an isotropic margin of 5 mm around the ITV.

The 4DMRI data were acquired at a 1.5 T MR scanner (*Magnetom Aera*, Siemens Healthcare, Erlangen, Germany), using a T1-weighted gradient echo sequence with radial stack-of-stars sampling and subsequent iterative 4D-reconstruction, based on a k-space-center self-gating signal [26]. A wooden flat table top was used at the MR scanner to ensure a reproducible positioning of the patient during imaging and treatment. The individual settings and parameters for the respective patients, as well as the time between the acquired 4DMRI scans can be found in Table 1. Up to six 4DMRI scans were acquired along the respective patient treatment course per patient.

**Table 1:** Number of available 4DMR images, patient positioning, beam angels of both fields (F0, F1), CTV volume and time between consecutive 4DMRI data sets

Patient	# 4DMRI	Positioning	Beam angle [°]		CTV volume [cc]	Time between consecutive 4DMRI scans [days]
			F0	F1		
P1	2	supine	160	210	51.9	1
P2	6	supine	160	210	33.0	15 / 5 / 9 / 3 / 10
P3	5	supine	160	210	95.1	6 / 9 / 5 / 11
P4	2	prone	20	340	87.8	21
P5	1	prone	20	340	194.8	–
P6	1	prone	20	340	115.1	–
P7	1	supine	160	200	112.0	–
P8	3	supine	20	340	62.4	15 / 24
P9	2	prone	20	340	46.0	7

The numbers of acquired 4DMRI scans per patients differed in our cohort since some patients were treated with carbon ions within 3 weeks whereas others had proton irradiations over 5–6 weeks and thus were available for more MRI scans. In addition, MR scanning time capacities did not allow a weekly MR scan in every patient.

All patients except P8 were treated with two oblique posterior beams. P8 was treated from anterior with only one field (340°), however, to allow a better comparison with the results for the other patients, a second field with 20° was included in the calculations for this patient.

For patients with less than five available 4DMRI data sets, the length of the breathing cycle of the available 4DMRI data was artificially varied between 3 and 10 s such as to generate different motion inputs (m) for the subsequent 4D dose calculation and thereby simulate day-to-day motion variations. The respective number of motion inputs (m) for each patient is listed in Table 2. For the simulated breathing cycles, parentheses are added in which the respective underlying input motion is given, e.g. 3.0 (m1) is the 4DMRI data set of measurement 1, where the breathing cycle was changed to be *T* = 3.0 s.

**Table 2:** Number of input motion patterns *m* for the 4D dose calculation and respective breathing cycles *T* [s] for P1-P9

	P1	P2	P3	P4	P5	P6	P7	P8	P9
*m1*	7.1	3.8	9.7	3.7	3.7	5.6	3.0 (m5)	4.7	3.7
*m2*	3.0 (m1)	2.8	8.2	3.0 (m1)	3.0 (m1)	3.0 (m1)	5.0 (m5)	3.0 (m1)	3.0 (m1)
*m3*	5.5 (m1)	3.7	8.8	5.5 (m1)	5.5 (m1)	5.5 (m1)	7.0 (m5)	10.0 (m1)	5.5 (m1)
*m4*	10.0 (m1)	3.7	8.4	8.0 (m1)	8.0 (m1)	8.0 (m1)	9.0 (m5)	5.6	2.9
*m5*	4.8	3.7	6.7	5.9	10.0 (m1)	10.0 (m1)	10.0	3.0 (m4)	3.0 (m4)
*m6*	3.0 (m5)	2.8		3.0 (m5)				10.0 (m4)	5.5 (m4)
*m7*	5.5 (m5)			5.5 (m5)				5.6	
*m8*	10.0 (m5)			10.0 (m5)				3.0 (m7)	
*m9*								10.0 (m7)	

### Synthetic 4DCT from 4DMRI

**Fig. 1:**
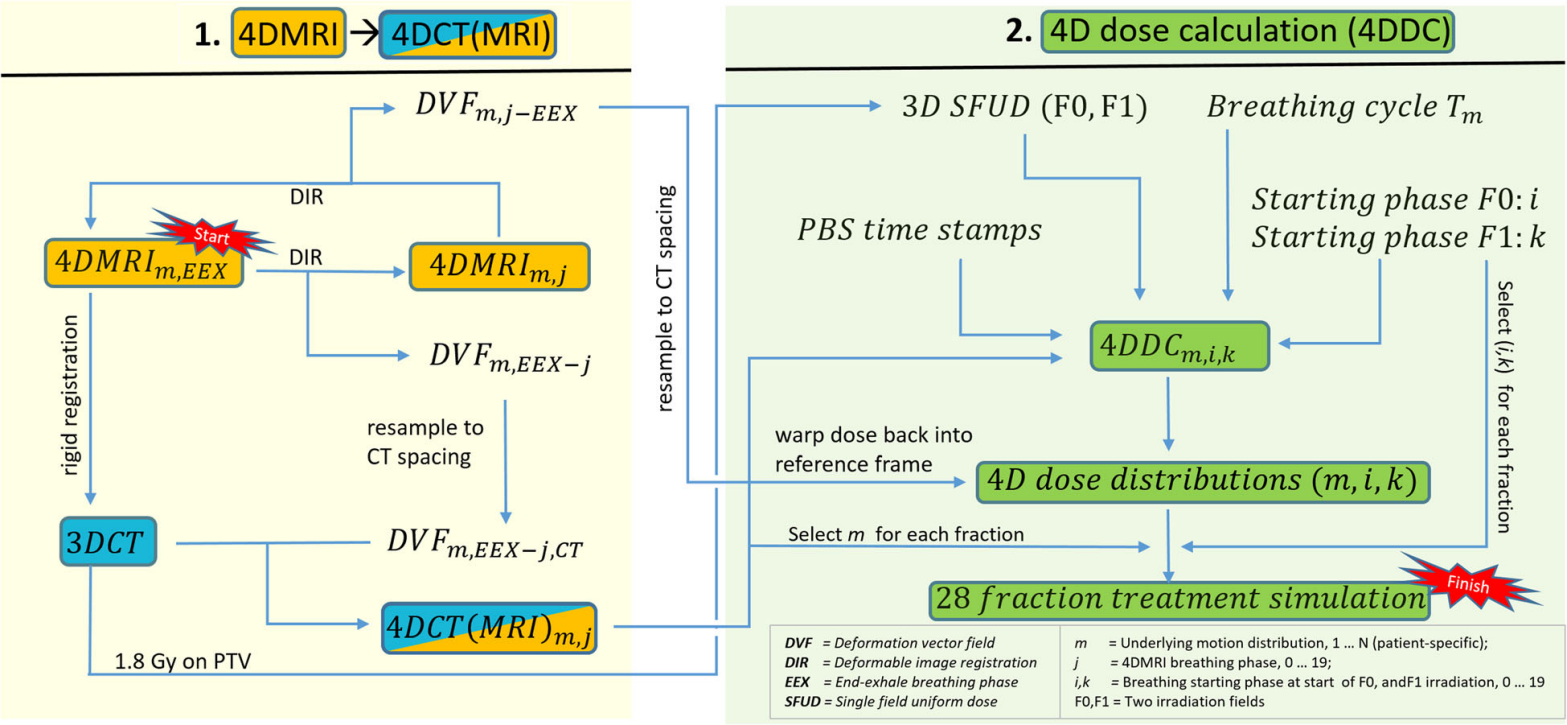
Schematic Workflow from 4DMRI via synthetic 4DCT(MRI) to 4D dose calculations for fractionated PBS proton therapy of pancreatic cancer

The workflow, starting from 4DMRI data acquisition to the final 4D dose calculations for fractionated PBS proton therapy, is illustrated in Fig. 1. In short, for each patient, synthetic 4DCTs, called 4DCT(MRI) in the following, were generated by warping a 3D treatment planning CT of the respective patient with deformation vector fields (DVFs), extracted from 4DMRI data. The end-exhalation breathing phase *EEX* of each 4DMR image was registered to all other breathing phases *j* of the same measurement (20 breathing phases in total) by means of deformable image registration (DIR), using the open-source software plastimatch (www.plastimatch.org) and the Demons algorithm [27]. In detail, the ITK implementation of the demons algorithm, using fast-symmetric-forces, was used with four different plastimatch stages and different scales of resolution and demons step lengths. The 3D planning CT was rigidly registered to the *EEX* MR image and the DVFs were used to deform the 3DCT to obtain a synthetic 4DCT(MRI) for each 4DMRI data set. The workflow is described in further detail in [23].

### CTV motion analysis and deformation field QA

The CTV motion was extracted from the 4DCT(MRI) by applying a binary CTV mask to the DVFs which results in motion distributions of all voxels within the CTV delineation for each breathing phase with respect to *EEX.*

For DIR quality assurance, Jacobian determinants were calculated inside the OAR delineations (liver, kidneys, bowel) for each 4DCT(MRI), using the DVF between the *EEX* and end-inhalation breathing phase *EIN* to evaluate volume conservation.

With respect to mass conservation, the delineated volume *V*_*EEX*_ of each OAR on the *EEX* phase of each 4DCT(MRI) was multiplied by the estimated physical density *ρ*_*EEX*_ for each voxel inside the delineation, obtained from a generic HU-to-density lookup table [28], to obtain the respective mass *m*_*EEX*_. Then, *m*_*EIN*_ = *V*_*EIN*_ · *ρ*_*EIN*_ was calculated with *V*_*EIN*_ being derived by warping the OAR delineation from *EEX* to *EIN* using the respective DVF and the respective density information *ρ*_*EIN*_. Finally, the ratio *m*_*EEX*_/*m*_*EIN*_ was considered as an estimate of mass conservation.

### Treatment planning and 4D dose calculation

For all patients, PBS proton therapy plans were used with 1.8 Gy (RBE) per fraction (RBE = 1.1) and 28 fractions, employing the beam angles of the treatments that had actually been delivered, see Table 1.

A 3D single field uniform dose (SFUD) plan with two fields for PBS proton therapy was calculated on the 3DCT for each patient. 4D dose calculations were performed by means of the PSI 4D pencil beam proton dose calculation algorithm [22, 29], taking into account the density changes, extracted from the 4DCT(MRI), the respective breathing cycle *T* for motion input *m* (see Table 1) and the beam dynamics of the PSI-Gantry2 [18], e.g. the time stamps and weights of each individual pencil beam. For each field, the 4D dose distributions for 20 different initial breathing starting phases of the patient, derived from the 20 available different breathing phases of each 4DMRI data set, were calculated.

For every fraction of the simulated 28 fraction PBS proton treatments, such a 4D dose distribution was calculated by random selection of both the underlying 4DCT(MRI)_*m*_ density information and the breathing starting phases *(i, k)* for both fields F0 and F1, see Fig. 1.

The resulting interplay effect was analyzed with respect to dose homogeneity in the CTV (*d5/d95*)*,* CTV coverage (*v95, v107*), mean dose *d*_*mean*_ and near maximum dose *d2* in the CTV for the 3D dose calculation (3DDC), as well as for the 4D dose distributions for single fractions (4Dx1) and 28 fractions (4Dx28), respectively. Here, *d5*, *d95* and *d2* denote the relative doses that 5, 95 and 2% of the volume receive, respectively, and *v95* and *v107* are the percentage volumes, that receive at least 95 and 107% of the prescribed dose.

Moreover, the dose-volume-histograms (DVHs) for OARs, namely left/right kidney, spinal cord, liver and bowel were analyzed with respect to *d*_*mean*_, *d2* and *v30*, where *v30* denotes the volume that receives at least 30% of the dose. The results were tested for significance by means of a single-sided Wilcoxon rank-sum test with a confidence level of 95% (*α* = 0.05).

### Correlation analysis

Inter-patient correlations between CTV motion amplitudes, length of breathing cycles and the interplay effect, assessed as d5/d95 of a single fraction, were determined by calculations of weighted correlations coefficients *ρ*_*w*_. The respective numbers of repeated 4DMRI data sets were used as weights for each patient, in compliance with previous publications [30, 31].

The statistical significance was calculated assuming a t-distribution of the data for small sample sizes with *N* = 9. In this case, the test statistic $t={\rho}_w\cdotp \sqrt{\left(N-2\right)/\left(1-{\rho}_w^2\right)}$ was calculated and compared to the tabulated critical value *t*_crit_ = 1.833 of the t-distribution for a one-sided test with a confidence level of 95%. Statistical significance was determined for *t > t*_crit_.

Furthermore, the correlation coefficient *ρ*_*m*_ between CTV volumes, which were considered constant for all fractions, and the mean interplay effects were calculated. The dependency of patient positioning (supine/prone) and beam angles on the interplay effect was analyzed by comparison of mean d5/d95 values. Additionally, for patients with multiple available 4DMRI data sets, intra-patient dependencies between CTV motion amplitudes, length of breathing cycles and the interplay effect (d5/d95) were analyzed.

### Longitudinal interplay investigation for a possible clinical application

In a possible clinical scenario, pre-treatment acquisitions of 4DMRI data sets could be used to estimate the statistical evolution of the interplay effect during fractionation. We investigated the uncertainty of such an estimation based on a single pre-treatment 4DMRI as opposed to multiple 4DMRI data sets acquired over the treatment course by comparing the respective evolution of the interplay effect, assessed as d5/d95. For all patients with ≥2 available 4DMRI scans (P1, P2, P3, P4, P8, P9), we performed 30 simulations each of the entire × 28 fractionated treatment scheme, taking into account only the first motion pattern (m1) to simulate a single pre-treatment 4DMRI acquisition, or all motion patterns, respectively. For the latter, for each treatment fraction the respective motion pattern was randomly sampled from the available patterns for each patient.

## Results

### Motion extraction from 4DMRI and vector field QA

**Fig. 2:**
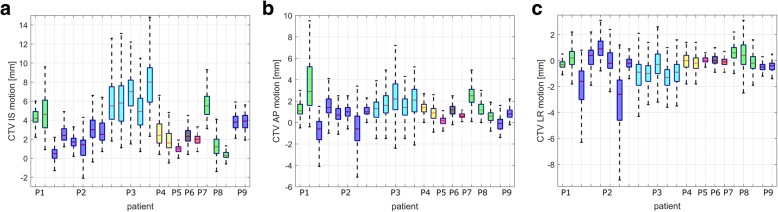
Motion distributions of all voxels inside the CTV delineation between end-inhale and end-exhale breathing phases for P1-P9 in **a** IS, **b** AP and **c** LR direction for the respective numbers of available 4DMRI data sets. The whiskers indicate the 95% range of the boxplots

The extracted CTV motion distributions in inferior-superior (IS), anterior-posterior (AP) and left-right (LR) direction are illustrated in Fig. 2 for all patients. They show the patient-specificity of the motion patterns as well as interfractional changes in CTV motion, extracted from the 4DCT(MRI). For this patient cohort, maximum CTV motion amplitudes of 15 mm were present with comparably small mean values of ≤8 mm in IS and ≤3 mm in AP and LR direction, respectively. Maximum intra-patient day-to-day differences in mean and maximum motion amplitudes of up to 5.3 mm and 8.1 mm (for P8), respectively, arose in IS direction, the dominant direction of motion.

The evaluation of volume and mass conservation yielded a mean Jacobian determinant among all patients of 1.00 ± 0.04, indicating volume-conserving DIR with low regional volume changes in the OARs. A mean mass conservation coefficient value of *m*_*EEX*_/*m*_*IN*_ = 1.07 ± 0.05 was derived.

### Static treatment plans

**Fig. 3:**
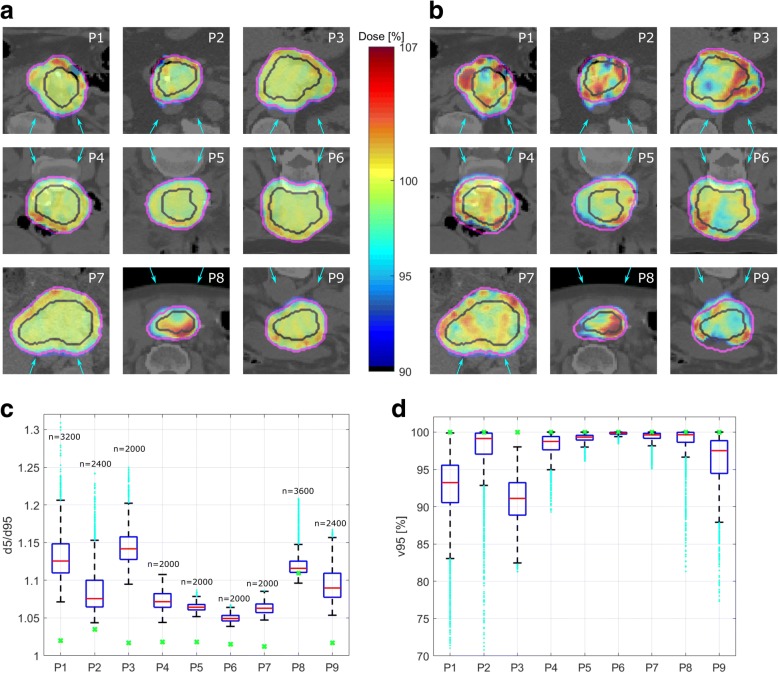
**a** 3D SFUD plans and **b** exemplary 4D dose distributions for single fraction PBS proton therapy (4Dx1) of patients P1-P9 with the respective delineations of CTV/PTV in black/pink. Doses below 90% of the prescribed dose are not displayed in this dose representation. The blue arrows indicate the beam angles. The interplay effect in terms of **c** homogeneity index *d5/d95* and **d** *v95* for all patients are displayed for *n* calculated possible 4D dose scenarios of single fraction treatments with variable initial breathing phases. The green crosses indicate the values of the static 3D case

The 3D SFUD plans revealed an average mean dose of *d*_*mean*_ *=* 99.6% (range 99.5–99.9%) with full CTV coverage (*v95* = 100%, *v107 = 0%*), high dose homogeneity with *d5/d95*≤1.03 (range 1.01–1.03) and near maximum dose values of *d2 < 103%* (range 100.3–103.0%) for 8 out of the 9 patients. For P8, slightly inferior values (*d*_*mean*_ *=* 101.4%, v95 = 100%, v107 = 6.5%, d5/d95 = 1.11, d2 = 108.9%) were obtained, as illustrated in Fig. 3a, c, d.

### 4D dose distributions for single fraction

By means of 4D dose calculations of a single fraction (4Dx1), the interplay effect of all combinations of initial breathing starting phases and underlying motion patterns was quantified in terms of *d5/d95* and *v95*. Depending on the patient, mean *d5/d95* = 1.05–1.14 were obtained with maximum values of up to 1.3. CTV coverage *v95* mean values of 92–100% were observed with minimum values of 70%, implying pronounced underdosage of the CTV. Moreover, near maximum doses *d2* of up to 110% were present within the CTV. These values show a strong dependency on the underlying motion input, which is discussed in detail in “Correlation analyses” section below. The calculated 3D SFUD plans, exemplary single fraction 4D dose distributions and single fraction interplay effects in terms of *d5/d95* and *v95* are displayed in Fig. 3. Pronounced hot and cold spots are visible in the 4D distributions.

### 4D dose distributions for 28 fractions treatment

**Fig. 4:**
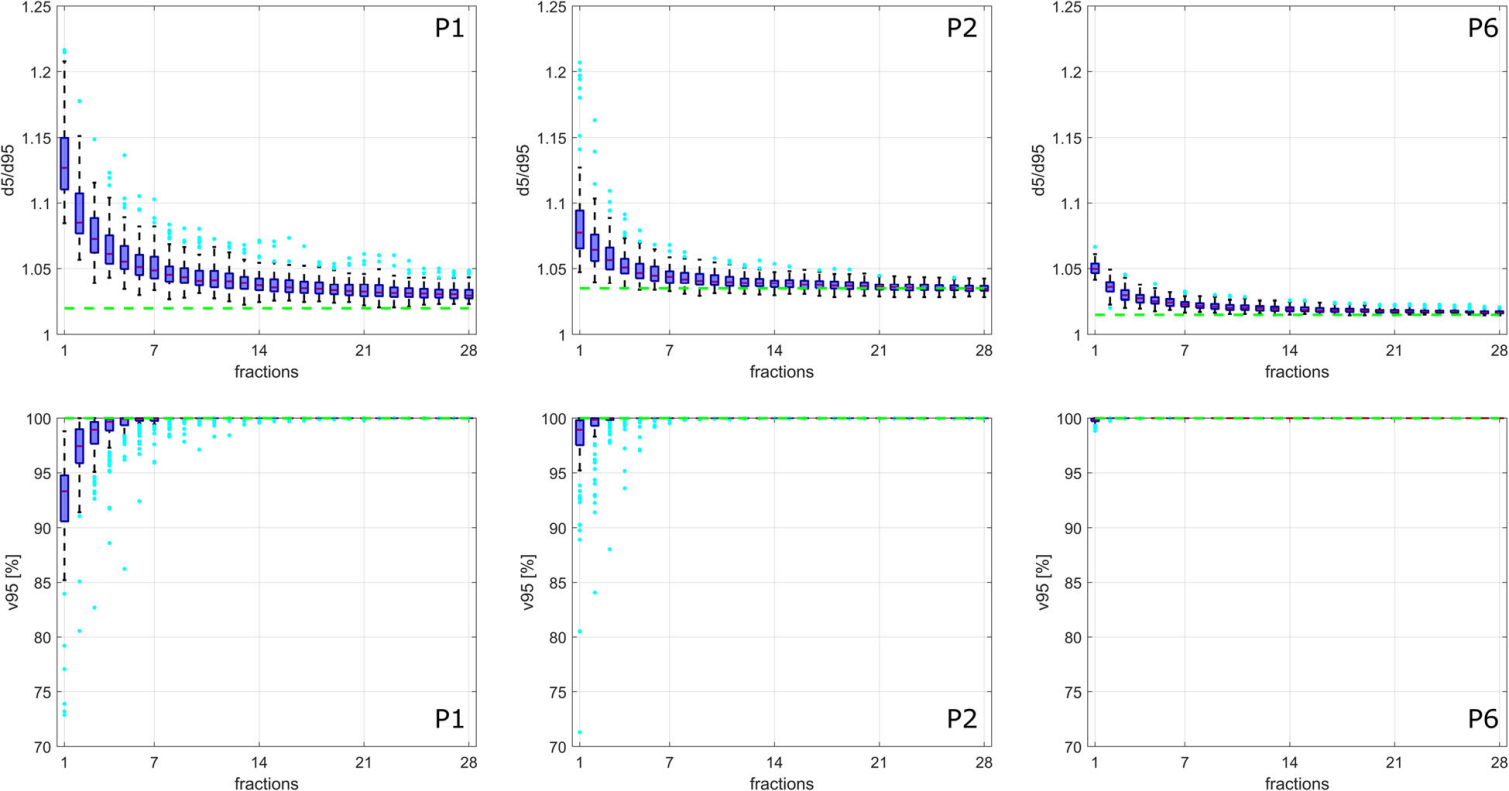
Exemplary *d5/d95* (**a**-**c**) and *v95* (**d**-**f**) distributions for 1–28 fractions PBS proton therapy of pancreatic cancer for P1/P2/P6, who showed a large/medium/small interplay effect. The green lines indicate the static 3D case

The simulated treatments with 28 fractions (4Dx28) show a patient-specific mitigation of the interplay effect as a function of the number of treatment fractions. Figure 4 displays example *d5/d95* and *v95* values for patients P1/P2/P6, who showed large/medium/small interplay effects in the single fraction calculations, respectively. Mean *d5/d95* decreased from 1.13/1.09/1.05 to 1.03/1.03/1.02 for these patients after 28 fractions, mean *v95* reaches 100% after 11/6/2 fractions, respectively.

We observed the same trend of an increased averaging effect of the interplay effect as a consequence of an increased number of fractions for all patients in this study. After a standard fractionation scheme with 28 fractions, the dose homogeneity *d5/d95* in the CTV was similar for both 4D and 3D dose distributions and *v95* = 100% was obtained for all patients after 2–14 fractions (mean: 6.7 fractions). Moreover, *d2* as well as *v107* were reduced by fractionation. Except for P8, *v107* = 0% was obtained after at most 10 fractions (mean: 5.6 fractions). For P8, *v107* = 6% was observed after 28 fractions.

**Fig. 5:**
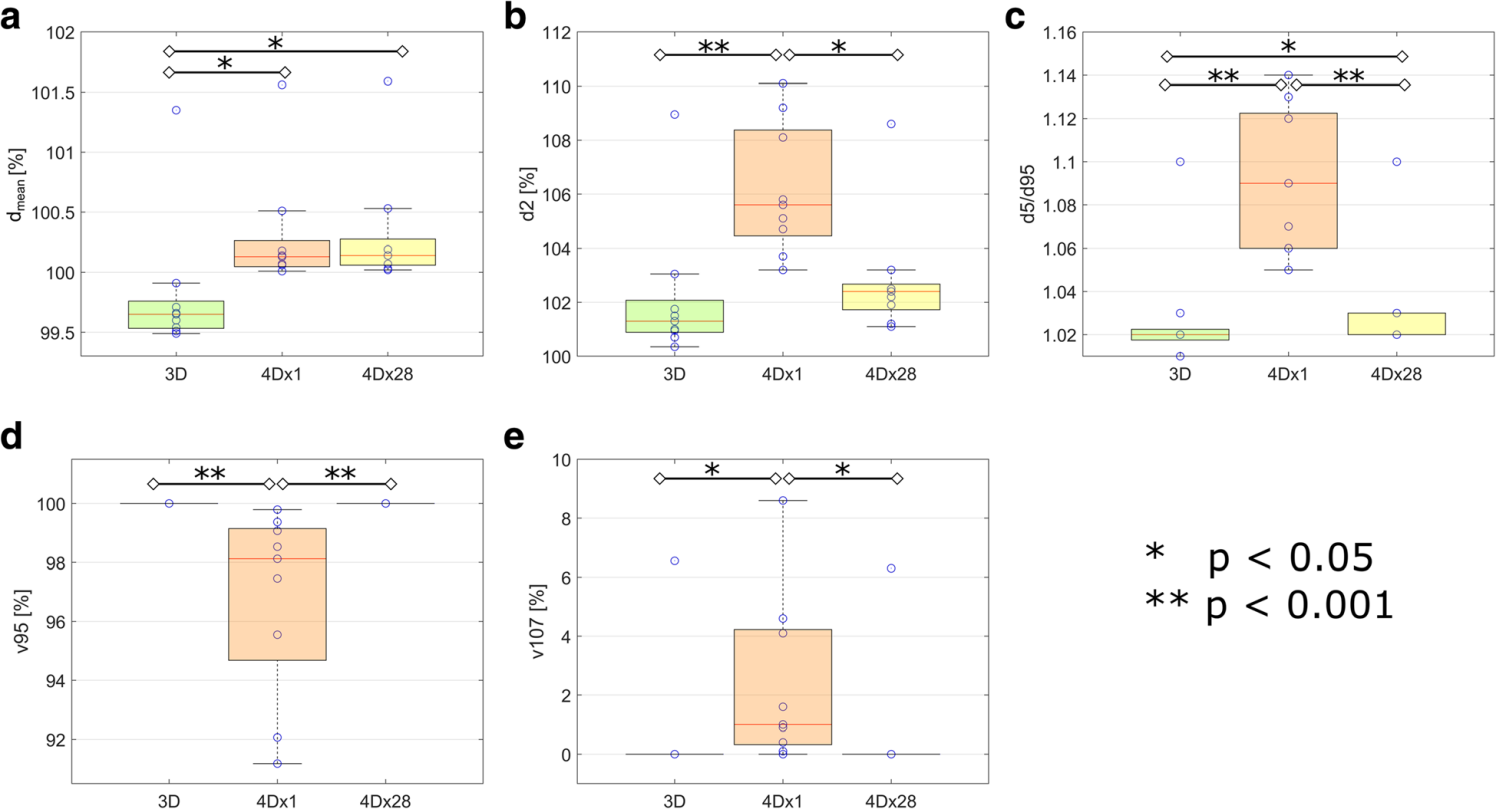
CTV dose quantities (*d*_*mean*_, *d2*, *d5/d95, v95, v107*) for P1-P9, resulting from 3D dose calculations (3DDC), as well as from 4D dose distributions for single (4Dx1) and 28 fractions (4Dx28), respectively

On average, *d*_*mean*_ increased by 0.47%/0.49% in the 4DDC for 1 or 28 fraction scenarios, respectively, compared to 3DDC. For *d5/d95, d2* and *v107*, we observed significantly higher values for 4Dx1 compared to both, 3DDC and 4Dx28, whilst *v95* was significantly lower for 4Dx1 compared to both 3DDC and 4Dx28, as apparent in Fig. 5.

With respect to d_*mean*_ and *d5/d95*, statistically significant differences between 3DDC and 4Dx28 were observed, which, however, appear to be clinically less relevant due to the small absolute differences (mean d_*mean*_ *=* 99.8% / 100.3% and mean *d5/d95 =* 1.028/1.034 for 3DDC/4Dx28*,* respectively). These results indicate that statistically for a large number of fractions, the interplay effect reduces to an acceptable CTV dose distribution in PBS proton therapy of pancreatic cancer.

### Dose to OARs

**Fig. 6:**
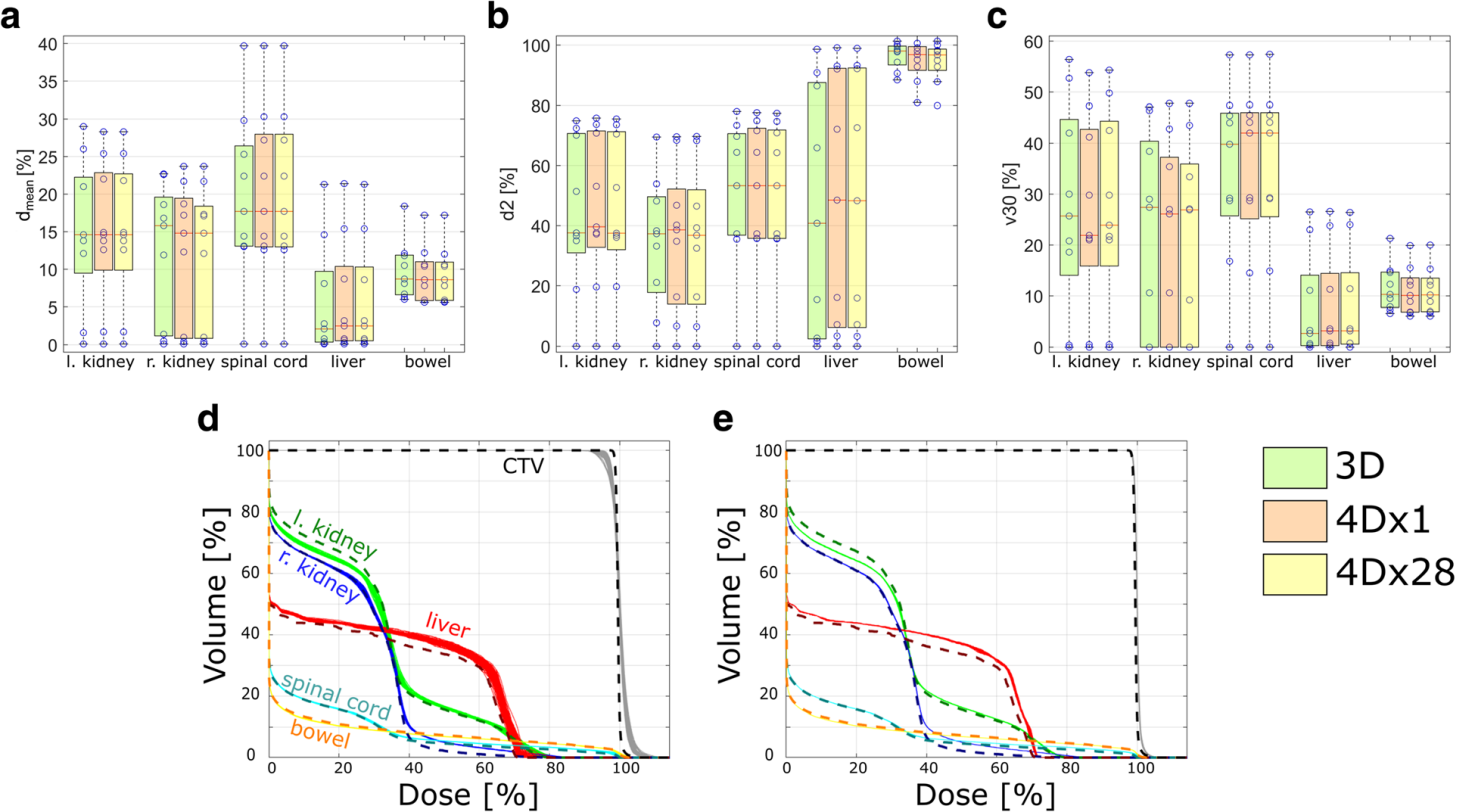
The OAR dose quantities **a** *d*_*mean*_, **b** *d2* and **c** *v30* show no significant differences when comparing static dose calculations (3DDC) with 4D scenarios with single (4Dx1) and 28 fractions (4Dx28)*.* Exemplary DVHs of P5 for **d** 4Dx1 and **e** 4Dx28 include both OARs and the CTV, as well as the respective dosimetric results from the 3D dose calculations in dashed lines

From the DVHs for the left/right kidney, spinal cord, liver and bowel, the dose quantities *d*_*mean*_, *d2* and *v30* were compared among 3DDC, 4Dx1 and 4Dx28. The deviations in *d*_*mean*_ were < 0.5% for all considered organs at risk. With respect to mean *v30*, deviations of < 1.2% were observed between the three different scenarios. The mean near maximum dose *d2* increased by 0.6–3.2% for all OARs (except in the bowel, where d2 decreased by 1.5–2.1%) when comparing 3DDC with both 4Dx1 and 4Dx28. Overall, no significant differences were found for the OARs, comparing the three different scenarios, see Fig. 6.

### Correlation analyses

**Fig. 7:**
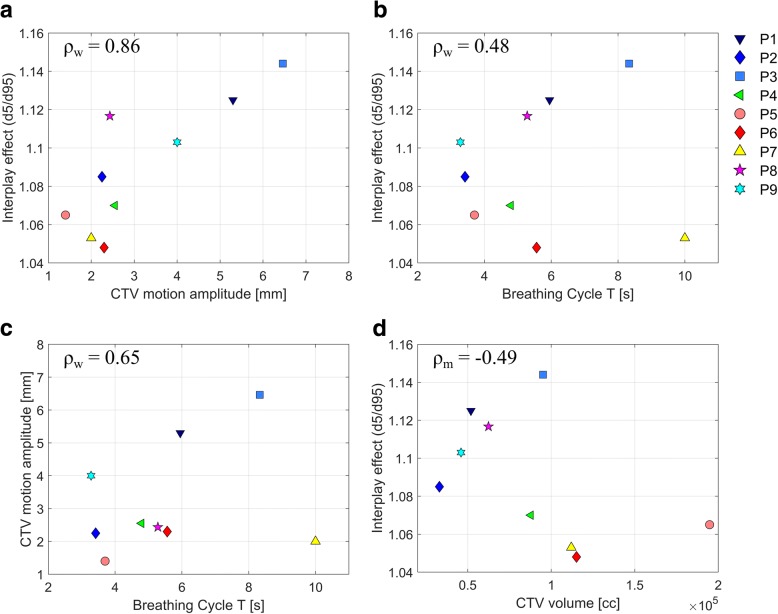
Inter-patient correlations between CTV motion amplitudes, length of breathing cycles and the interplay effect assessed as d5/d95 (**a**-**c**). For each patient, the mean values of the respective quantities are displayed. The correlation between the interplay effect and the CTV volume is illustrated in (**d**)

The analysis of inter-patient correlations showed significant (t > t_crit_ = 1.833) positive correlations between CTV motion amplitudes on the one hand, and the length of breathing cycles and the interplay effects assessed in terms of d5/d95 on the other hand, as illustrated in Fig. 7. The strongest correlation was revealed between the CTV motion amplitudes and *d5/d95* (*ρ*_*w*_ = 0.86, *t* = 4.46). Smaller, but still significant correlations were found between breathing cycle and CTV motion amplitude (*ρ*_*w*_ = 0.65, *t* = 2.26). The correlations between the length of breathing cycles and d5/d95 (*ρ*_*w*_ = 0.48, *t* = 1.45) and between CTV volume and d5/d95 (*ρ*_*m*_ =  − 0.49, *t* = 1.49) were determined to be not significant*.*

Patients in supine positioning (mean d5/d95 = 1.10) showed a similar magnitude of interplay effect as patients in prone positioning (mean d5/d95 = 1.08).

The intra-patient analysis revealed patient-specific both proportional and anti-proportional dependencies between CTV motion amplitude, interplay effect and length of breathing cycles with no clear tendency. Due to the small sample sizes, no correlation coefficients were determined.

### Longitudinal interplay investigation towards a possible clinical application

**Fig. 8:**
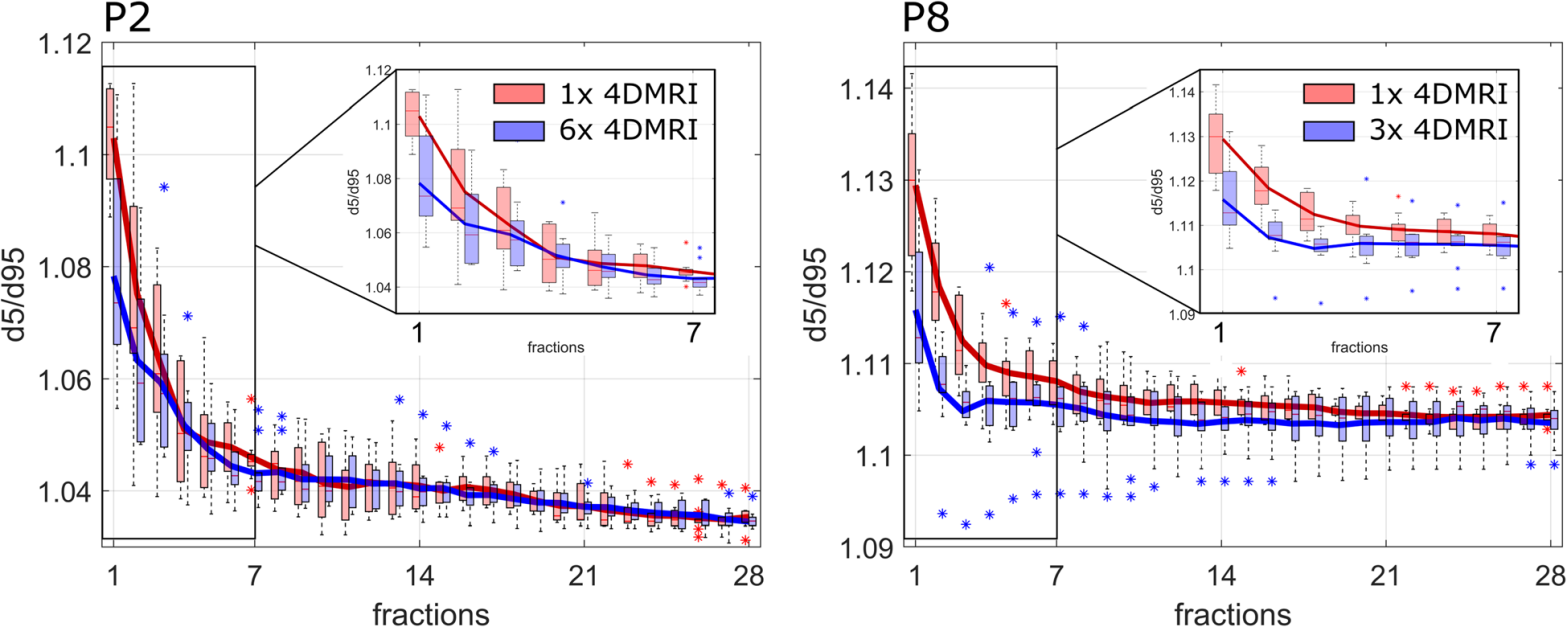
Estimation of the statistical evolution of the interplay effect based on a single 4DMRI (red) compared to 5/3 4DMRI scans for P2/P8 (blue), respectively, for 30 simulated treatments. In the multi-4DMRI scenarios, the underlying motion pattern was randomly sampled for each fraction. The solid lines represent the respective mean d5/d95 values

For a pre-treatment estimation of the statistical evolution of the interplay effect during a × 28 fractionated treatment scheme, we found that a single 4DMRI scan may be sufficient for patients with low day-to-day motion variations, which was the case in this study (day-to-day variations of mean motion amplitudes ≤5.3 mm ). Due to the interplay mitigation effect by variable initial breathing phases in different fractions, after 28 fractions the mean d5/d95 value in 4D simulations with a single underlying 4DMRI or all available 4DMRI scans for the respective patients differed only by 0.1%. For smaller numbers of fractions (< 5), the estimation of the interplay effect by the single-4DMRI approach differed by up to 3% compared to the multiple-4DMRI approach. Figure 8 illustrates both scenarios for P2 and P8.

## Discussion

This study shows a pronounced averaging effect of the interplay effect by means of fractionation for a cohort of 9 pancreatic cancer patients. On average, after 7 fractions (range 2–14), a sufficient CTV coverage of 95–107% was obtained. This result is especially interesting with respect to hypofractionated treatments, as for instance performed in a Japanese carbon-ion dose escalation study with 8 fractions, which showed nevertheless promising outcomes [32]. However, although fractionation leads to a mitigation of the interplay effect, still in every single fraction hot and cold spots occur, whose clinical impact should be evaluated separately. For hypofractionated treatments or large motion amplitudes, additional motion mitigation could for instance be achieved by means of abdominal corsets [33, 34]. Alternatively, the choice of suitable small gating windows could further improve the resulting dose distributions and is of interest for further studies.

With respect to correlations, we found strong and medium positive inter-patient correlations between the CTV motion amplitudes on the one side and both the length of breathing cycles and the interplay effect on the other side, which is in good agreement with Dowdell et al., who found similar correlation for the interplay effect in lung proton treatments [35]. We are aware that our patient cohort shows comparably low CTV motion amplitudes, whereas pancreas motion amplitudes of > 30 mm have been reported [36]. From the observed correlations between CTV motion amplitudes and the amount of interplay effect, we therefore expect a stronger impact of the interplay effect for large-motion patients than for our patient cohort.

We need to acknowledge several limitation in our study here. First, deformable dose mapping is generally limited by uncertainties resulting from the performed image registrations and the resulting VFs [37, 38]. The VF-QA, performed in this study, showed sufficient Jacobian determinant values of 1.00 ± 0.04. In terms of mass conservation, it has been pointed out, that deformable image registrations in general may show certain uncertainties concerning mass conservation, since CT Hounsfield Units may be averaged during transformation [39]. We observed relative mass differences in the considered OARs between the *EIN* and *EEX* images of 7 ± 5%. Similar mean deviations of 5 ± 7% have been reported for DIR of thoracic CTs with deviations up to 50% [40], which illustrate the intrinsic uncertainties of deformable image registration.

Second, the demonstrated method of warping a static CT by means of various 4DMRI vector fields holds further intrinsic limitations. Geometrical differences in the patient anatomy between different days, such as translations of pancreas due to gastrointestinal distention [41], are not fully considered in this 4D treatment planning approach, as the same patient CT is deformed for all of the various motion inputs of the same patient. Moreover, the free-breathing treatment planning CTs were registered to the EEX phase of the respective 4DMR images, which visually showed a better agreement to the CT than the mid-ventilation MR images. However, a detailed statistical analysis to determine the most suitable of all available 20 breathing phases of each 4DMRI could further reduce the registration uncertainties. Alternatively, a more challenging deformable multi-modal CT-MR deformable image registration could be considered in future studies.

Furthermore, only regular breathing motion was investigated. The impact of irregular motion pattern on the interplay mitigation should be investigated in future studies. For the simulated 4D data sets we did not change the motion amplitudes when varying the length of breathing cycles due to the unclear intra-patient correlations between CTV motion amplitudes and length of breathing cycles. However, it is unclear whether the amplitudes would indeed be the same for different lengths of breathing cycles, which is a limitation of this study.

Finally, we would like to rephrase the importance and advantages of using repeated 4DMRI data sets of patients for subsequent 4DCT(MRI) generation and 4D dose calculation, compared to the conventional 4DCT approach. Being an imaging modality without any imaging dose to the patient, regular 4DMRI data could be acquired from the patients, saving 20–200 mGy compared to a single 4DCT measurement [42]. By means of 4DMRI data, the duration of data acquisition may be extended over multiple breathing cycles, which provides more information than a single 4DCT snapshot of a single breathing cycle.

Previous studies have shown that pancreas motion shows huge day-to-day variations and one single 4DCT may not be representative for the tumor motion during a treatment [43], lasting over several weeks. Repeated 4DMRI measurements, acquired prior to the treatment, can help to observe these motion variations and could potentially be used for robust analyses or worst-case optimizations [44], based on real patient-specific motion patterns. In future scenarios, if online 4DMRI is available during irradiation on hybrid MR-Linac devices [45–48] or possible MR-proton devices, such an approach can also be used for online 4DMRI-based dose recalculations. For instance, static MR images could be acquired prior to each fraction to account for geometrical differences and patient setup errors. Then, 4DMRI could be acquired simultaneously during each irradiation fraction and the derived vector fields could be used to retrospectively calculate a 4D dose distribution of the day by means of the proposed method in this study and to accumulate all 4D fraction doses.

Even without online MR image-guidance, we found that our proposed method to quantify the interplay effect as a function of fractionation provides a viable tool for pre-treatment estimations of the statistical evolution of the interplay effect within an uncertainty range of a few percent for patients with low day-to-day motion variations. This is due to the fact, that variable initial breathing phases during irradiation fractions have a larger impact on the mitigation effect than small differences in breathing motion patterns. Therefore, such an approach allows an estimation of the statistical mitigation of the interplay effect by means of pre-treatment 4DMRI acquisitions. Based on such pre-treatment 4DMRI data, criteria may be defined to determine, whether additional motion limitation by abdominal compression or the application of gating criteria may be required for a specific patient.

## Conclusion

We found 4D dose evaluation, based on repeated 4DMRI data sets, to be a promising methodology to investigate the mitigation of the interplay effect in PBS proton therapy of pancreatic cancer treatments. 4D investigations of a patient cohort of 9 pancreatic cancer patients with CTV motion amplitudes < 15 mm showed significant positive correlations between CTV motion amplitudes one the one hand and both the interplay effect and the length of breathing cycles on the other hand. We observed a gradually increased mitigation of the interplay effect with an increased number of fractions. After an average of 7 fractions, a sufficient CTV coverage of 95–107% was observed and the dose homogeneity within the CTV was similar in 3D and 4D dose distributions. For hypofractionated treatments, the study indicates a more pronounced impact of the interplay effect for pancreatic cancer treatments. For patients with low day-to-day motion variations, such as those enrolled in this study, the statistical evolution of the interplay effect along the treatment course could be estimated based on a single 4DMRI.

## Abbreviations

3D: Volumetric
3DDC: 3D dose calculation
4D: Time-resolved volumetric
4DCT(MRI): Synthetic 4DCT, generated from 4DMRI
4DDC: 4D dose calculation
4Dx1: 4D dose distribution for a single fraction
4Dx28: 4D dose distribution for 28 fractions
AP: Anterior-posterior
CT: Computed tomography
CTV: Clinical target volume
d2, d5, d95: Relative dose, that 2%/5%/95% of the volume receive
DIR: Deformable image registration
DVF: Deformation vector field
DVH: Dose-volume-histogram
EEX: End-exhalation breathing phase
EIN: End-inhalation breathing phase
GTV: Gross tumor volume
IMRT: Intensity-modulated radiation therapy
IS: Inferior-superior
ITV: Internal target volume
MRI: Magnetic resonance imaging
OAR: Organ at risk
PBS: Pencil beam scanning
PTV: Planning target volume
QA: Quality assurance
RT: Radiation therapy
SFUD: Single field uniform dose
v30, v95, v107: Volume, that receives 30%/95%/107% of the prescribed dose
